# In silico analysis of TUBA4A mutations in Amyotrophic Lateral Sclerosis to define mechanisms of microtubule disintegration

**DOI:** 10.1038/s41598-023-28381-x

**Published:** 2023-02-06

**Authors:** Akshatha Ganne, Meenakshisundaram Balasubramaniam, Haarika Ayyadevara, Lily Kiaei, Robert J. Shmookler Reis, Kottayil I. Varughese, Mahmoud Kiaei

**Affiliations:** 1 Department of Geriatrics, University of Arkansas for Medical Sciences, Little Rock, AR 72205 USA; 2 Central Arkansas Veterans Healthcare Service, McClellan Veterans Medical Center, Little Rock, AR 72205 USA; 3 SiBioLead, LLC, Little Rock, AR 72207 USA; 4 University of Arkansas, Fayetteville, Fayetteville, AR 72701 USA; 5 University of California, Los Angeles, Los Angeles, CA 90095 USA; 6 RockGen Therapeutics, LLC, Little Rock, AR 72205 USA; 7 Department of Physiology and Cell Biology, University of Arkansas for Medical Sciences, Little Rock, AR 72205 USA; 8 Department of Pharmacology and Toxicology, University of Arkansas for Medical Sciences, Little Rock, AR 72205 USA; 9 Department of Neurology, University of Arkansas for Medical Sciences, 4301 W. Markham St., Slot 611 (BioMed 1, Rm B-306A), Little Rock, AR 72205 USA

**Keywords:** Drug discovery, Neuroscience, Structural biology, Neurology

## Abstract

Amyotrophic lateral sclerosis (ALS) is an inexorably progressive and degenerative disorder of motor neurons with no currently-known cure. Studies to determine the mechanism of neurotoxicity and the impact of ALS-linked mutations (*SOD1*, *FUS*, *TARDP*, *C9ORF72*, *PFN1*, *TUBA4A* and others) have greatly expanded our knowledge of ALS disease mechanisms and have helped to identify potential targets for ALS therapy. Cellular pathologies (e.g., aggregation of mutant forms of SOD1, TDP43, FUS, Ubiqulin2, PFN1, and C9ORF72), mitochondrial dysfunction, neuroinflammation, and oxidative damage are major pathways implicated in ALS. Nevertheless, the selective vulnerability of motor neurons remains unexplained. The importance of tubulins for long-axon infrastructure, and the special morphology and function of motor neurons, underscore the central role of the cytoskeleton. The recent linkage of mutations to the tubulin α chain, TUBA4A, to familial and sporadic cases of ALS provides a new investigative opportunity to shed light on both mechanisms of ALS and the vulnerability of motor neurons. In the current study we investigate TUBA4A, a structural microtubule protein with mutations causal to familial ALS, using molecular-dynamic (MD) modeling of protein structure to predict the effects of each mutation and its overall impact on GTP binding, chain stability, tubulin assembly, and aggregation propensity. These studies predict that each of the reported mutations will cause notable structural changes to the TUBA4A (α chain) tertiary protein structure, adversely affecting its physical properties and functions. Molecular docking and MD simulations indicate certain α chain mutations (e.g. K430N, R215C, and W407X) may cause structural deviations that impair GTP binding, and plausibly prevent or destabilize tubulin polymerization. Furthermore, several mutations (including R320C and K430N) confer a significant increase in predicted aggregation propensity of TUBA4A mutants relative to wild-type. Taken together, these in silico modeling studies predict structural perturbations and disruption of GTP binding, culminating in failure to form a stable tubulin heterocomplex, which may furnish an important pathogenic mechanism to trigger motor neuron degeneration in ALS.

## Introduction

Amyotrophic lateral sclerosis (ALS) is a fatal disease resulting from progressive loss of dendrites, axons, and neuromuscular junctions leading to the degeneration of motor neurons. The mechanisms leading to selective motor-neuron loss in ALS are not fully understood. Axonal integrity and structural/functional specialization of motor neurons may create vulnerabilities to neuropathology, further impacted by mutations to genes with roles in axonal maintenance and function. The cytoskeleton is critical to viability and function of all cells, and may play an especially important role in neurons due to their special requirements for axonal transport and intercellular connectivity. Microtubules, which form the structural framework of all cells, comprise heterodimers of tubulin chains. Mutations impacting the molecules that create these complex structures are expected to often disrupt the function and survival of neurons. Thus far, mutations to at least seven tubulin-family members have been linked to diverse neurodevelopmental and neurodegenerative disorders^1^, and mutations of multiple genes, viz., *SOD1*, *TDP43*, *FUS/TLS*, *C9orf72*, *PFN1*, *TUBA4A*, *TANK1*^2^ were specifically linked to familial ALS (fALS). Studies conducted on these genetic linkages provided valuable information bearing on the pathogenic mechanism(s) of motor-neuron degeneration^3^. Moreover, a study of exome rare alleles implicated variants in *tuba4a* (encoding a tubulin α chain) as causal factors for fALS^4^, and *tuba4a* variants predicted to be structurally disruptive were also significantly enriched in sporadic ALS cases^5^. These mutations are considered to be pathological, and were hypothesized to perturb microtubule dynamics and stability^6^, although empirical studies confirming this have yet to appear. At present, little is known regarding mechanisms by which single-amino-acid changes impair axonal integrity and function, leading to motor-neuron degeneration as observed in ALS.

To elucidate how *tuba4a* mutations lead to ALS and other neurodegenerative diseases, we used computational methods to predict how *tuba4a* mutations linked to ALS might impact tubulin structure and polymerization, to the detriment of motor neuron viability, axonal transport, or the neuronal cytoskeleton. Alpha (α) and beta (β)-tubulin heterodimers are the basic building blocks that polymerize to assemble microtubules. TUBA4A is one of the two major tubulins of microtubules, suggesting cytoskeletal dysfunction as a possible mechanism for ALS. On the other hand, mutations in genes encoding tubulin subunits are linked to a broad spectrum of human neurological diseases^7^ and conditions referred to as “tubulinopathies”^8^. Mutations of tubulin beta 4A (TUBB4A) have also been reported in hypomyelinating leukodystrophy diseases^9,10^.

Tubulins form the principal subunits of microtubules, which are essential determinants of cell morphology, motility, and rigidity; they are also indispensable to antero/retrograde transport along neuronal axons. Six out of eight mutations in TUBA4A (G43V, R215C, R320C, R320H, A383T and W407X) are suggested to be pathogenic or disease-contributory mutations^4^. However, we currently have no insight into how, or how severely, each mutation impacts axonal integrity, microtubule assembly, stability and function. The current study focuses on mutations, including R320C and R320H, which are associated with fALS families with disease duration of 1–3 years. Other studies have provided evidence to support the hypothesis that *TUBA4A* mutations can be pathogenic for ALS^5,6,11^. Diverse tubulin mutations have been linked to other human diseases; e.g., mutations in the TUBA4A auto-regulatory domain (R2G heterozygotes) were linked to dystonia type 4, associated with Wilson’s disease^12^, whereas mutation in TUBA1A causes Lissencephaly in humans^13^. We hypothesize that tubulin-gene mutations may lead to intrinsic protein misfolding, causing disorders associated with dysregulated neurogenesis and degeneration of previously differentiated neurons.

Landers and Shaw described eight TUBA4A variants in ALS patients and presented data suggesting that these atypical α-tubulins impact the dynamics of microtubules^4^. Therefore, changes in the amino acid sequence of TUBA4A (whether point mutations or truncation of this protein at its C-terminus) likely disrupt the formation of complex quaternary structures and act as the principal mechanism for motor neuron dysfunction and mortality.

In general, GTP binds at the interface between TUBA4A and TUBB4A^14^. Experimental structures of tubulin heterodimer complexed with GTP reveal that GTP interacts with TUBA4A in the heterodimer complex, at two glycine-rich sites known as the E (exchangeable) and N (non-exchangeable) sites^15^. Experimental studies showed that GTP binding to the heterodimer complex is a crucial step in tubulin polymerization^16^.

We hypothesize that modifications to the tubulin genes may lead to structural changes that modify the folding and/or ligand-binding properties of the encoded proteins, thus impairing microtubule assembly, cell morphology, and axonal transport.

## Results

### Molecular docking of GTP to wildtype and mutant tubulin heterodimers

**Figure 1:**
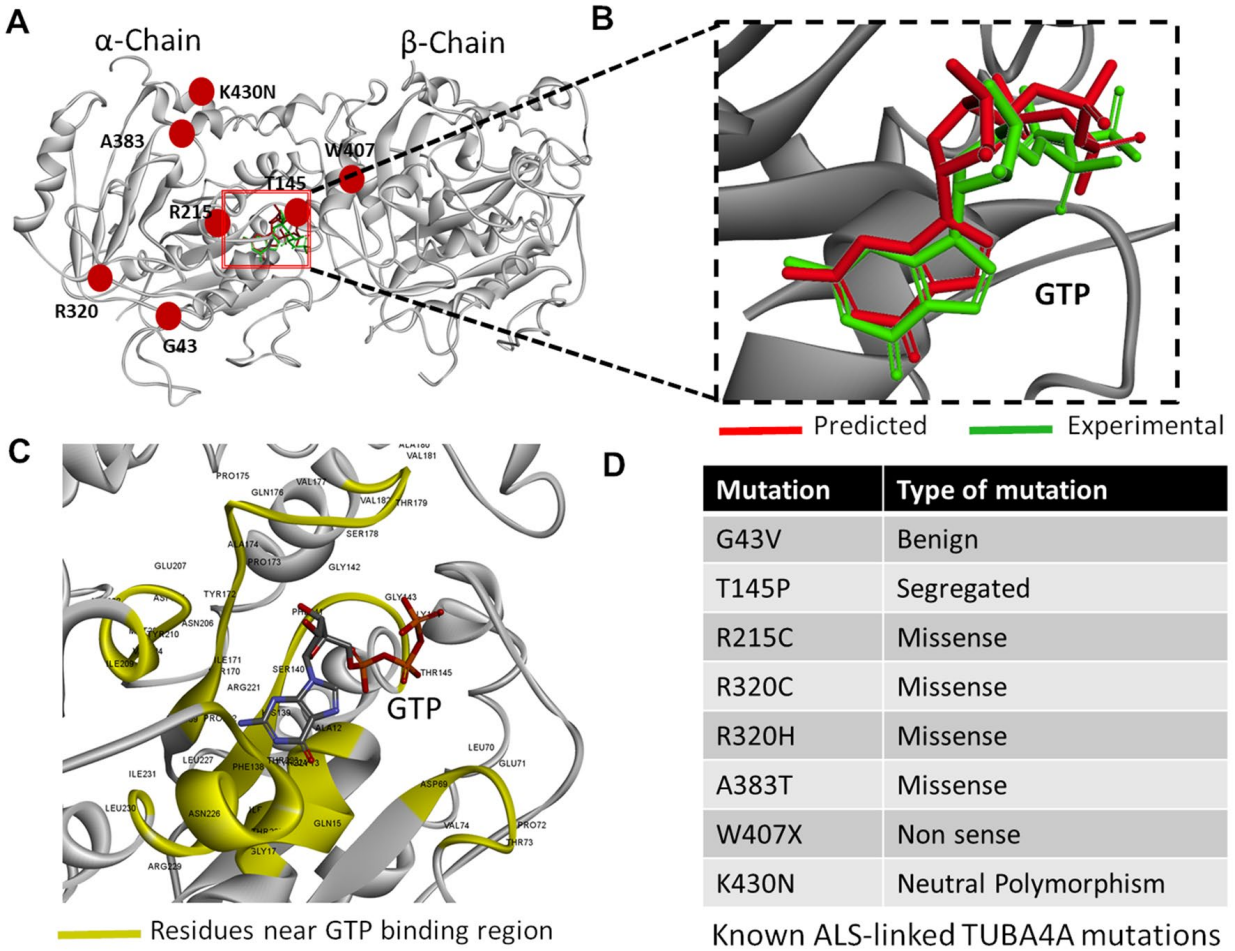
Molecular docking is consistent with experimental models of GTP binding by tubulin dimers. (**A**) Structure of tubulin α:β dimer, with GTP at the interface (PDB 1FFX). (**B**) GTP binding poses at the tubulin-dimer interface are nearly identical for in silico prediction (red) vs. crystallography data (green). (**C**) TUBA4A residues comprising the GTP binding pocket, which may interact directly with GTP, are highlighted in yellow. (**D**) Eight TUBA4A mutations are listed that are associated with familial ALS^3^.

To determine the structural impact of each TUBA4A mutation associated with fALS^4,17^, we first isolated the wild-type α-tubulin (TUBA4A) full-length structure from the heterodimeric crystal structure in the Protein Data Bank (PDB-ID: 1FFX) as an initial structure. Since this structure has missing loops and side-chains, we created a full-length TUBA4A structure using fold-recognition and ab initio structure-prediction methods from the ITASSER server. The heterodimeric complex consists of tubulin α-chain (TUBA4A) and tubulin β-chain (TUBB4A), complexed with GTP at their interface (Fig. 1A,B). TUBA4A has a molecular weight of 55 kDa and comprises 448 amino acid residues; TUBB4A has a molecular weight of 50 kDa, comprising 444 amino acids. Previous experimental studies demonstrated that certain TUBA4A mutations, linked to specific neuronal and oligodendrocyte defects, can impair microtubule integrity^7^. We sought novel insights into the impact of each mutation on microtubule formation and integrity by predicting their effects on protein structure, GTP binding, and dimerization with TUBB4A (β chain). We first docked GTP to the wild-type tubulin heterodimer complex as a reference control (Fig. 1A–C), using a standard protocol. To achieve robust prediction models, docking was conducted in parallel with Glide and Autodock-Vina algorithms to implement flexible-ligand docking. Both programs produced results consistent with the experimental structure, 1FFX, in that orientations and binding sites differ very little between predicted (red) *vs*. experimental (green) GTP poses (Fig. 1B).

### Molecular-dynamic simulations predict structural changes with tubulin mutations

**Figure 2:**
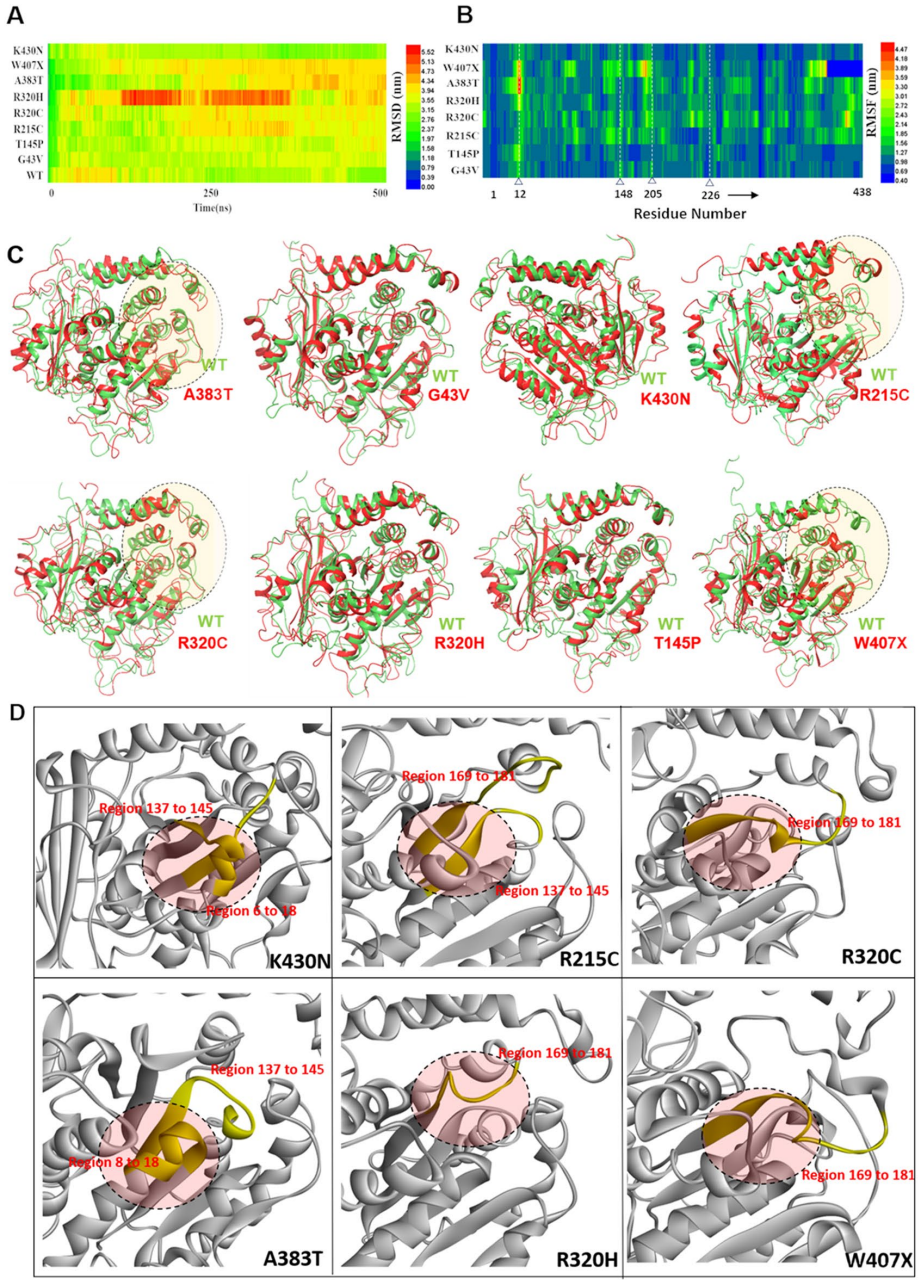
Atomistic molecular-dynamic simulations of mutated tubulins predict their structural effects. (**A**) Heat maps represent Root Mean Square Deviation (RMSD) of wild-type tubulin α chain and 8 mutants (rare alleles) that reveal differences in structural stability over time. Higher RMSD values (red and orange) indicate greater structural changes, while blue and green represent the least change. **B**) Heat maps represent Root Mean Square Fluctuation (RMSF) across TUBA4A at residues 1–432, averaged over the course of 500-ns simulations. Numbered triangles indicate GTP-contact residues referenced in the text. All values (indicated in the key at panel right) are normalized to wild-type α tubulin (not shown; represented by deep blue-green, 0.98). (**C**) Molecular-structure diagrams at the final (500-ns) frame of α-tubulin mutant simulations (red) are superimposed on the wild-type structure (green), to depict structural perturbations due to mutations. The most notable structural changes are highlighted within shaded dashed ovals in mutants A383T, R215C, R320C and W407X. (**D**) Enlarged structural diagrams of GTP-binding regions for 6 TUBA4A mutations (with GTP-interacting residues highlighted in yellow).

In order to model the effects of 8 mutations reported to be associated with ALS (A383T, G43V, K430N, T145P, R215C, R320H, R320C, and W407X) (Fig. 1D) on TUBA4A protein structure and function, we used computational modelling and atomistic molecular-dynamic simulation tools to analyze TUBA4A::TUBB4A dimerization, GTP binding, and microtubule assembly and stability. Beginning with the wild-type, full-length monomeric TUBA4A structure, we introduced amino acid substitutions or (for W407X) truncation corresponding to these mutations (see Methods for details). These monomeric TUBA4A mutated structures were simulated for 500 nanoseconds (ns) using the Desmond simulation package to explore and model the effects of introducing TUBA4A mutations relative to wild-type. TUBA4A comprises a folded core flanked by two intrinsically disordered tails that are “hotspots” for post-translational modification^18^. Since post-translational modifications, especially phosphorylations, play crucial roles in protein function and aggregation, analyzing perturbations in such regions is informative. These simulations show moderate to high structural destabilization by the ALS-associated mutations. The Root Mean Square Deviation (RMSD) of TUBA4A monomer, calculated from 500-ns simulation trajectories, was substantially elevated for 7 of the 8 mutant structures relative to wild-type TUBA4A (Fig. 2A). Because wild-type TUBA4A itself is partially disordered, its RMSD does not appear to reach a stable plateau during this simulation (Fig. 2A). Several of the mutant TUBA4A structures (G43V, R320H, and A383T in particular, but also R215C, R320C, and W407X) showed high RMSD fluctuation toward the end of each simulation, relative to fluctuations noted in WT. One mutant structure, T145P, was predicted to deviate less than wild-type.

RMSD reflects predicted structural deviation from the initial structure, whereas RMSF (Root Mean Square Fluctuation) displays the average predicted fluctuation of each residue over the full course of the 500-ns simulation, here normalized to the WT structure (Fig. 2B). The RMSF prediction for α-tubulin mutant W407X shows high fluctuation near the center and at the C-terminus of the truncated protein (Fig. 2B). Fluctuations of all other mutant proteins are distributed across the length of the alpha chain, with an average fluctuation of about 2.7 Å. Some of these fluctuating regions include GTP-binding residues (positions 12, 148, 205, and 226), indicated by triangles in Fig. 2B. In order to understand perturbations in the overall secondary structures, we calculated percentage structural changes for α-helix and β-sheets for target mutations compared to wild-type. These models indicate no significant changes to the overall TUBA4A secondary structures predicted for the mutated monomers (Fig. S1A). The average RMSD of all frames in the 500-ns simulation was calculated and plotted (Fig. S1B).

Since we took the final conformation after a 500-ns simulation as our starting structure for further computational modeling, we compared the RMSD of the final frame with the mean RMSD of all frames in 500-ns simulations with respect to each mutation. In each case, the RMSD of the final conformation differed by < 1 Å from the mean RMSD (Fig. S1B). However, superimposing the simulated final conformation of the mutated structures with the wild-type structure shows that almost all of the mutations considered here perturb the structure at certain critical regions (Fig. 2C). Since GTP binding is crucial in maintaining the stability of tubulin polymer, we focused on the structural changes near the GTP-binding pocket (ovals in Fig. 2C). Significant structural perturbations were apparent in the GTP-binding region, and in the dimerization regions of mutants R215C, R320C, A383T, and W407X (Fig. 2C). Closer inspection of the GTP-binding regions of TUBA4A mutated structures reveals critical structural changes over residues 6–18 and 137–145 of both K430N and A383T mutant chains, whereas folding of residues 169–181 was altered in mutants R320H and W407X (Fig. 2D). As these regions, viz., 6–18, 137–145, and 169–181, are part of the GTP binding region in TUBA4A, our analyses predict that TUBA4A mutations R215C, R320H/C, A383T, and W407X are likely to impact GTP binding and/or dimerization, whereas G43V should not elicit major structural changes in either region (Fig. 2C,D).

### Molecular docking predicts impaired GTP binding for several α-tubulin/TUBA4A mutations

Because simulation analyses predicted mutation-induced structural perturbations, especially at the TUBA4A GTP-binding region, we hypothesized that certain mutations (including W407X, R215C, and A383T) may deter GTP binding. The ability of tubulin monomers to self-assemble into large polymers in the presence of GTP, and to disassemble upon GTP hydrolysis, is an essential characteristic of microtubule dynamics, and is critical to many biological processes such as cell motility, mitotic chromosome segregation, and establishment of cell polarity^19^. Disruption of GTP binding can prevent tubulin polymerization and may lead to microtubule disassembly^20^, leading to apoptosis^21^. Since GTP binds at the interface between α and β tubulins, we compared its binding energy to wild-type vs. mutant heterodimers. For this purpose, we replaced wild-type tubulin α chain in the tubulin α:β/GTP crystal structure (PDB 1FFX), with the 500-ns simulated structures of mutant tubulin α chains, to form mutated heterodimeric complexes. A detailed flow chart for the generation and analysis of mutant heterodimers is presented in Fig. S2. The resulting structures were briefly simulated in a solvent box using GROMACS, to resolve any transient instabilities arising from chain replacement. GTP docking was followed by molecular mechanics based on generalized Born and surface area (MM-GBSA) solvation calculations to predict the Gibbs binding free energy (ΔG_binding_) of GTP to wild-type and mutant heterodimers.

**Figure 3:**
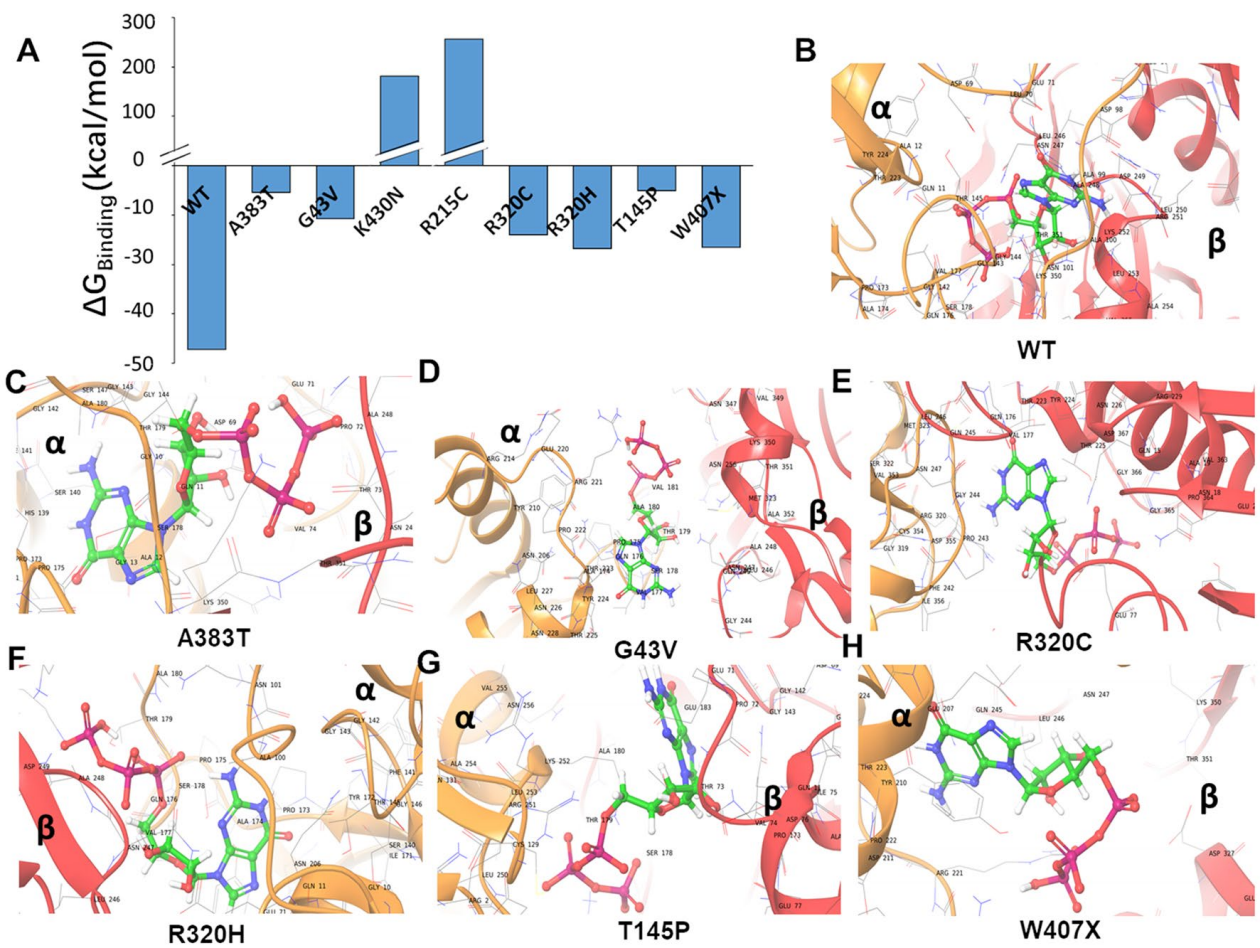
Molecular docking predicts impaired GTP binding. (**A**) Molecular docking and MM-GBSA binding free energy calculations, for GTP binding by wild-type (WT) and mutated tubulin heterodimers (comprising WT or mutant α chains, heterodimerized with wild-type β chain). The GTP binding energy (ΔG_binding_) was weakened by all 8 mutations, and was completely eliminated by K430N and R215C. (**B**–**H**) Binding pose predictions show perturbed GTP binding to tubulin α:β heterodimers containing fALS-associated tubulin α-chain mutations.

Our results predict that the stability of GTP binding to wild-type heterodimer (ΔG = ‒ 47 kcal/mol) would be severely weakened by six of the α chain mutations, which reduced the predicted ΔG_binding_ by 60‒85%, whereas K430N and R215C heterodimers could not bind GTP at all (Fig. 3A) since that would require a considerable energy expenditure (> 150 kCal/mol). Binding-pose predictions imply that G43V, a relatively benign mutation, would allow GTP to bind in a position and orientation similar to those of wild-type dimer (compare panels B and D of Fig. 3), despite low binding affinity. Binding poses predicted for A383T, R320C, R320H, and T145P indicate GTP binding orientations quite different from that of wild-type complex (Fig. 3B‒H), which may further disrupt microtubule polymerization beyond what is expected based only on ΔG estimates.

### MD simulations of tubulin α:β/GTP complexes predict dimer destabilization by α mutants

**Figure 4:**
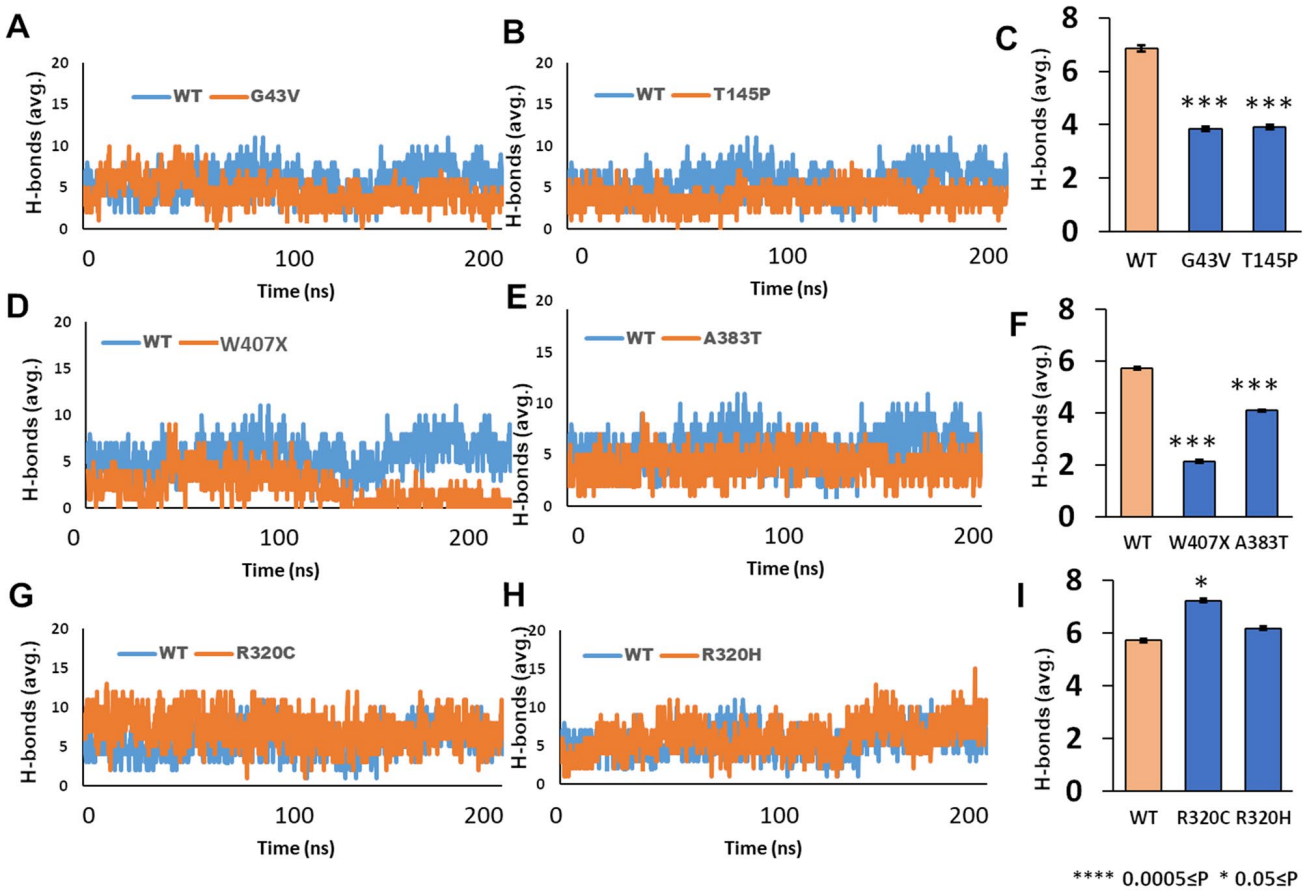
MD simulations predict destabilization of mutated tubulin α:β heterodimers with bound GTP. (**A**, **B**, **D**, **E**, **G**, **H**) Calculated intermolecular hydrogen bonds linking wild-type tubulin β chain to wild-type (blue) or mutant (orange) tubulin α chain, over 200-ns molecular-dynamic simulations of α:β heterodimers. (**C**, **F**, **I**) Histograms summarize average hydrogen-bond totals connecting WT or mutant α chains to wild-type β chain during the last 50 ns of each simulation.

Since previous studies reported that certain TUBA4A mutations impaired microtubule stability^4^, we used 200-ns molecular-dynamic simulations to predict the effect of each observed TUBA4A mutation on the stability of dimer complexes with GTP bound at the dimer interface. As noted in the preceding section, dimers containing TUBA4A mutations K430N and R215C could not be simulated in this way because they do not bind GTP spontaneously (Fig. 3), and this conclusion was supported by the failure of Glide docking to produce any binding pose. We plotted intermolecular hydrogen bonds between α and β tubulin chains, using trajectory analysis across a 200-ns time course (Fig. 4). Mutation W407X was predicted to reduce H-bonding throughout the simulation, trending toward zero H bonds over the final 80 ns (Fig. 4D), whereas mutants G43V, T145P, and A383T only dipped below control levels for the final 50 ns (Fig. 4A–F). Two of the mutations (R320C and R320H) had no discernible effect on dimer integrity, with interchain hydrogen-bond (H-bond) trajectories similar to wild-type (Fig. 4G–I). Taken together, these data predict a wide range of mutational effects on tubulin α:β heterodimer stability. The Root Mean Square Deviation (RMSD), and Root Mean Square Fluctuation (RMSF) of mutant dimers showed significant departures from wild-type, suggesting that the mutations disrupt dimer stability (Figs. S3 and S4).

### TUBA4A mutations aggravate aggregation propensity

**Figure 5:**
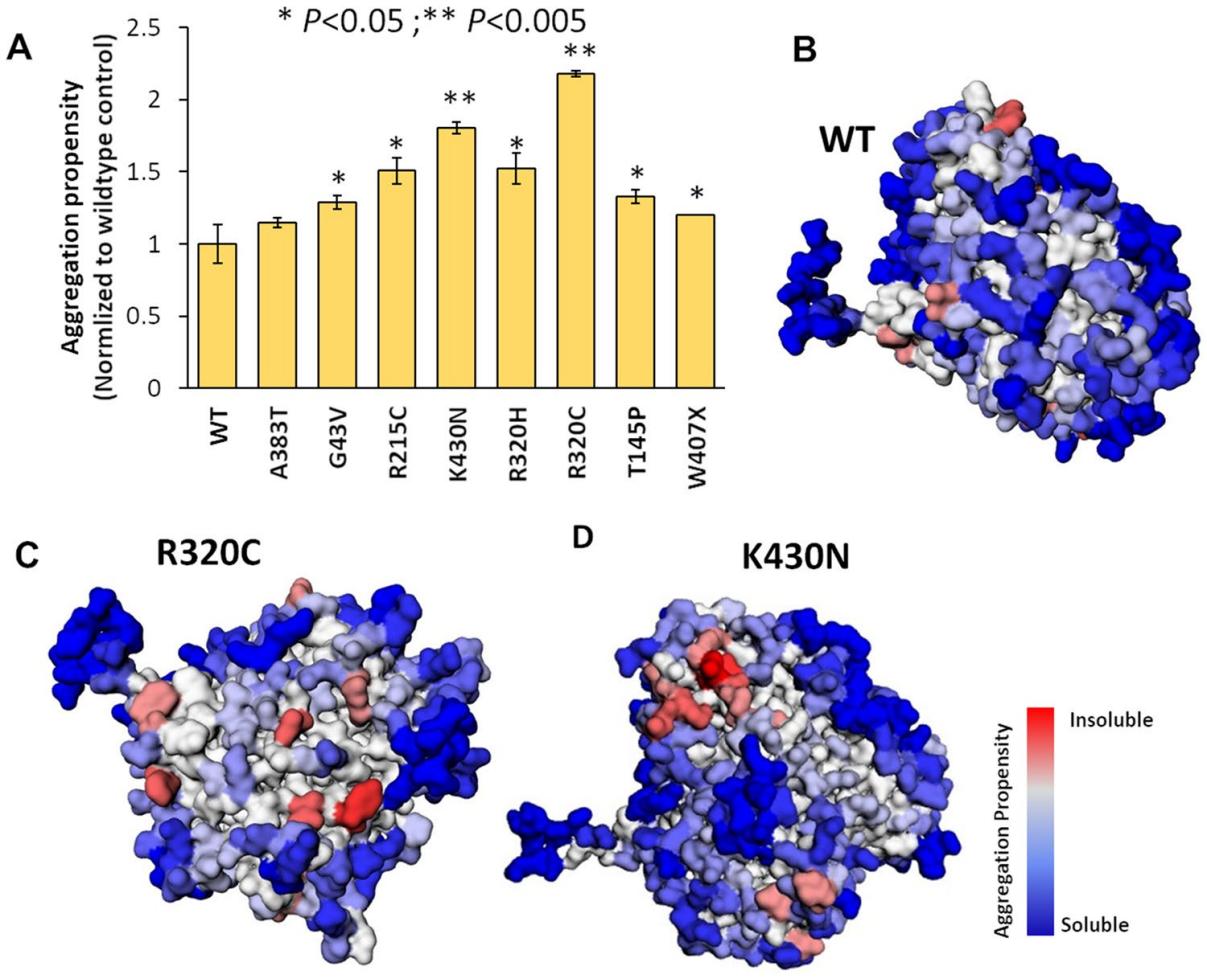
Structural analysis using Aggrescan3D to predict aggregation propensity of wild-type vs. mutant alleles of tubulin α chain. (**A**) Histogram summarizing aggregation propensity scores for each mutation, normalized to wild-type α tubulin. (**B**‒**D**) Structural diagrams of wild-type vs. mutant (R320C and K430N) α tubulin chains, with predicted aggregation propensity hotspots indicated by the color key ranging from red (highest propensity) to blue (lowest aggregation propensity, or highest hydrophilicity). Although all mutants have somewhat higher aggregation propensity than wild-type, R320C and K430N show the greatest exposure of highly hydrophobic regions.

Misfolding, insolubility, oligomerization, and aggregation of proteins are pathological hallmarks of ALS, and other neurodegenerative diseases^22^. Proteomic analysis of insoluble aggregates identified several tubulin isoforms specific to Alzheimer patient brains^22^. MD simulations of these mutated TUBA4A structures predicted substantial unfolding of tubulin, which may increase susceptibility to aggregation. We therefore calculated aggregation propensity for the simulated (500 ns) TUBA4A structures, wild-type and mutated, to predict whether structural perturbations arising from these ALS-linked mutations may predispose them to coalesce with other proteins found in ALS-specific inclusion bodies. Aggrescan3D (ver. 2.0) is a multi-parameter tool to predict aggregation likelihood from simulations of flexible protein structures. Aggregation scores, calculated for each mutant-dimer structure, were normalized to that of the wild-type dimer. The K430N and R320C mutations confer 1.8- and 2.2-fold higher aggregation propensity, respectively, than the wild-type α chain (Fig. 5), consistent with previous experimental results^4^. The R320H mutation is predicted to be less inclined to aggregation than R320C, while other mutations conferred still lower (but nevertheless significant) increases in aggregation propensity (Fig. 5A). In Fig. 5, panels B–D illustrate the greater exposure of hydrophobic (pink to red) and moderately insoluble (grey to light blue) residues on the surface of the TUBA4A chain, for the two mutants deemed most likely to aggregate.

## Discussion

Cytoskeletal dysfunction is increasingly recognized as a pathogenically important feature of neurodegenerative diseases such as ALS^23,24^; in particular, mutations in genes that encode critical cytoskeleton proteins (e.g. dynactin, profilin1, TUBA4A) have been linked to familial ALS (fALS)^7,11,17^. In this study, we performed unbiased structural modeling and atomistic molecular-dynamic simulations to predict structural consequences of tubulin α chain (TUBA4A) mutations identified in fALS pedigrees by exome-wide sequencing^4^. Such rare alleles typically represent established population variants that are inherited at higher frequency than new mutations arise. Biochemical studies, documenting the impact of observed mutations on tubulin polymerization and on increased aggregation propensities^4^, are consistent with our in silico findings. However, the present study provides structural insights into the mechanisms whereby single-amino-acid substitutions in the TUBA4A sequence can exert broad impacts on GTP binding, dimer stability, and susceptibility to aggregation. Microsecond (0.5 µsec) MD simulations modeled the dynamic effects of structural alterations in crucial regions including the GTP binding site, for a set of TUBA4A variants reported as fALS-linked. Despite the dynamic and partially disordered structure of tubulin monomers, the microtubule comprising co-polymerized tubulin α and β chains is the most rigid cellular polymer known^17^. Glutamates are highly represented in both α and β tubulin tails, the regions with the highest propensities for intrinsic disorder; aspartate residues are also present, contributing to the electronegativity of these regions^17^. Reported TUBA4A mutations examined in this in silico work were predicted to disrupt GTP binding relative to wild-type TUBA4A:TUBB4A dimers. Binding of GTP at the interface of TUBA4A::TUBB4A is crucial for the polymerization, growth and stability of microtubules^25^; our prediction that the reported mutations could directly affect GTP binding suggests, at least in part, an explanation for the etiology of ALS pathology.

Analysis of dimer stability via MD simulations predicted the greatest disruptive effects for mutations G43V, A383T, T145P, and W407X—all of which are expected to reduce dimer integrity and hydrogen bond count (Fig. 4). These predictions support, and are entirely consistent with, a previous experimental study that showed disruption of microtubule polymerization by most of the same mutations^4^. We focused on the impact of reported mutations on GTP-dependent tubulin polymerization. For example, the G43V mutation produced relatively little impact on the stability of tubulin heterodimers (Fig. 4C), consistent with the clinical description of this mutation as benign^4^. Nevertheless, the severity of pathology could also be affected by interactions of other factors involved in the maintenance of microtubule integrity, which may compensate for a mild loss of GTP binding and/or of inherent dimer stability. In contrast, the W407X mutation is a nonsense (truncating) mutation, a detrimental or pathological allele leading to a drastic reduction in microtubule polymerization. Aggregation propensity is another property exacerbated by almost all TUBA4A mutations, which might contribute to pathogenicity despite remaining largely independent of effects on dimer stability or GTP binding. Smith et al.^4^ showed a detrimental effect of the W407X mutation, associated with formation of multiple aggregate-like inclusions. Although aggregation propensity based on protein structure was predicted to increase only modestly for the *W407X* nonsense mutation, the truncated protein it encodes might evade protein degradation machinery to form aggregate inclusions as noted by Smith et al.^4^. Mutations R320C and K430N conferred the greatest increases in predicted aggregation propensities. Structural analyses implicated exposed hydrophobic regions as potential sites of intra-aggregate adhesion (Fig. 5B‒D).

Functional studies showed that the R320C α-tubulin mutant, after transient overexpression in HEK293 cells, was particularly defective in generating α/β tubulin dimers^4^. The R320 residue of TUBA4A is conserved from metazoa to humans, implying that it plays a critical role in determining the structure and function of α-tubulin, likely reflecting the importance of arginine’s positive charge and relative bulk. Substitution of cysteine for arginine may impact tubulin interactions with motor proteins, including kinesin^26^, leading to abnormalities in transport and trafficking. This may impact, at least in part, the R320C phenotype in lysosomal transport and trafficking, vesicle formation, and mitochondrial function.

For the present in silico investigation, computational tools were applied to each pathogenic TUBA4A mutant to predict changes at the atomic level that could impact interactions within the α subunit, at the dimerization interface between α and β tubulin chains, or within the GTP-binding pocket. These simulations suggest plausible mechanisms to account for pathogenicity of mutant α chains, leading to motor-neuron degeneration typical of ALS. For instance, the R320C mutation is of particular interest because of its linkage to fALS pedigrees with relatively short disease survival^4^. In vitro overexpression of a TUBA4A cDNA carrying R320C suggested problems with microtubule assembly and disruption of axonal anterograde/retrograde transport, which may impair motor neuronal functions^4^. The R320C mutation may not only disrupt microtubule assembly by disfavoring polymerization of tubulin subunits, but might also impede tubulin interactions with motor molecules such as kinesin, block acetylation of α-tubulin^27^, and/or create dysfunctional disulfide bridges. Our proposed models predict that R320C would bind GTP poorly, and would form unstable dimers.

Structural models based on molecular-dynamic simulations provide insights into structural perturbations in tubulin structure with respect to reported mutations. Even though we have not included biochemical analysis in this report, our extensive computational analysis strongly indicates that the mutations affect dimer stability and GTP binding consistent with previously reported experimental evidence, suggesting that these models may be of value to drug discovery for treating specific fALS mutations.

## Materials and methods

### Generation of mutated structures and their atomistic molecular-dynamic simulations

The reference heterodimeric tubulin structure, comprising tubulin α (TUBA4A) bound to β (TUBB4A) chains (PDB-ID 1FFX), was retrieved from the Protein Data Bank repository (www.rcsb.org). Since this available crystal structure contains gaps (missing atoms and residues), the full-length structure of TUBA4A was computationally modelled by fold recognition and ab initio methods, using the ITASSER server (https://zhanglab.dcmb.med.umich.edu/I-TASSER/). The predicted heterodimeric structure was then dynamically simulated for 10 ns using WebGRO^28^ (https://simlab.uams.edu) in a fully solvated box. The final simulated structure was the starting point for all mutational studies, into which TUBA4A amino acid substitutions were introduced using the Schrödinger Maestro mutagenesis module. Before simulations, mutated structures were preprocessed using the Protein Preparation Wizard from Schrödinger Maestro’s Prime Module^29,30^. Monomeric TUBA4A protein was immersed in an orthorhombic box containing Simple Point Charge (SPC) water along with NaCl sufficient to provide counterions for charged residues. To achieve quasi-physiological conditions, a further 0.15-M NaCl was added to the system. Prior to the actual MD run, the simulation system was energy-minimized and equilibrated as previously explained^30^. Atomistic molecular dynamic simulations were conducted for 500 ns using the Desmond simulation package^31^.

Structures of wild-type and mutated heterodimeric complexes were taken as the final frames from 500-ns simulations of full-length TUBA4A structures bearing various mutations, exchanged with the wild-type α chain of the reference heterodimeric complex (PDBID: 1FFX) using Coot™ software. Newly generated heterodimeric complexes were briefly simulated using the GROMACS simulation package, after which GTP and GDP were added and the heterodimer complexes (protein and GTP/GDP complex) were simulated using the Desmond simulation package which were immersed in an orthorhombic box containing Simple Point Charge (SPC) water along with NaCl sufficient to provide counterions for charged residues. To achieve quasi-physiological conditions, a further 0.15-M NaCl was added to the system.

### MD simulation trajectory analysis

Trajectory analyses for all simulations were generated using the Simulation Interaction Diagram module from Desmond Maestro suite. To assess reproducibility, simulations were repeated three times for monomeric simulations and twice for dimeric simulations. Results from replicate repeats were averaged, and the mean values were used to plot heat maps of root mean square deviation (RMSD) over time, and root mean square fluctuation (RMSF) at each residue.

### Glide-Grid generation and ligand docking of GTP to tubulin monomers and dimers

The Glide docking program was used for all protein–ligand dockings. A docking grid box was created around the GTP-binding region, enclosing the TUBA4A::TUBB4A interface and including all GTP-binding residues within 20 Å of their centroid, consistent with previous MD simulation studies of macromolecular complexes^32^. After the GTP ligand was prepared using the LigPrep wizard (Schrodinger Suite, Maestro), Glide docking was performed in Standard Precision mode. The Prime MM-GBSA module was then used to estimate the binding free energy (ΔG) of GTP binding^33^. GTP docking to mutant dimers using the Autodock-Vina package was conducted using SiBDock from https://sibiolead.com/^34,35^.

### Prediction of aggregation propensity

Aggregation propensity for mutated dimers was predicted by Aggrescan 3D 2.0, a Python-based program, utilizing protein 3-dimensional structures and stability in “dynamic mode”; these scores were then normalized to the wild-type complex.

### Prediction of hydrogen bond formation

The number of inter-molecular hydrogen bonds expected between tubulin α and β chains was estimated from all simulation frames, using the VMD Hydrogen Bond plugin.

## Supplementary Information

Supplementary Legends.

Supplementary Figure S1.

Supplementary Figure S2.

Supplementary Figure S3.

Supplementary Figure S4.

## Data Availability

The protein structure files predicted in the current study are available in the Mendeley Data Repository, https://data.mendeley.com/datasets/yk6dyys48w/1.
