# Supplementary material for: In silico analysis of TUBA4A mutations in Amyotrophic Lateral Sclerosis to define mechanisms of microtubule disintegration
Source: Sci Rep. 2023 Feb 6;13:2096. doi: 10.1038/s41598-023-28381-x (PMC9902468; doi:10.1038/s41598-023-28381-x)
Supplement: Supplementary file 1 — Supplementary Legends. [file 41598_2023_28381_MOESM1_ESM.pdf]

## Supplementary Figure Legends

**Figure S1.** Predicted secondary structure changes with respect to mutant TBB4A monomers based on 500ns molecular dynamics simulations.

**Figure S2.** Flow chart depicting the modeling work proposed in the manuscript.

**Figure S3**. RMSD plots for tubulin α:β heterodimers, contrasting wild-type (blue) to 8 α-mutated (orange) structures. Note changes in scale (y axis).

**Figure S4**. RMSF plots for tubulin α:β heterodimers, contrasting wild-type (blue) to 8 α-mutated (orange) structures. Regions showing the greatest mutant-WT differences are highlighted with red rectangles.
