# Supplementary figures and images for: In silico analysis of TUBA4A mutations in Amyotrophic Lateral Sclerosis to define mechanisms of microtubule disintegration
Source: Sci Rep. 2023 Feb 6;13:2096. doi: 10.1038/s41598-023-28381-x (PMC9902468; doi:10.1038/s41598-023-28381-x)

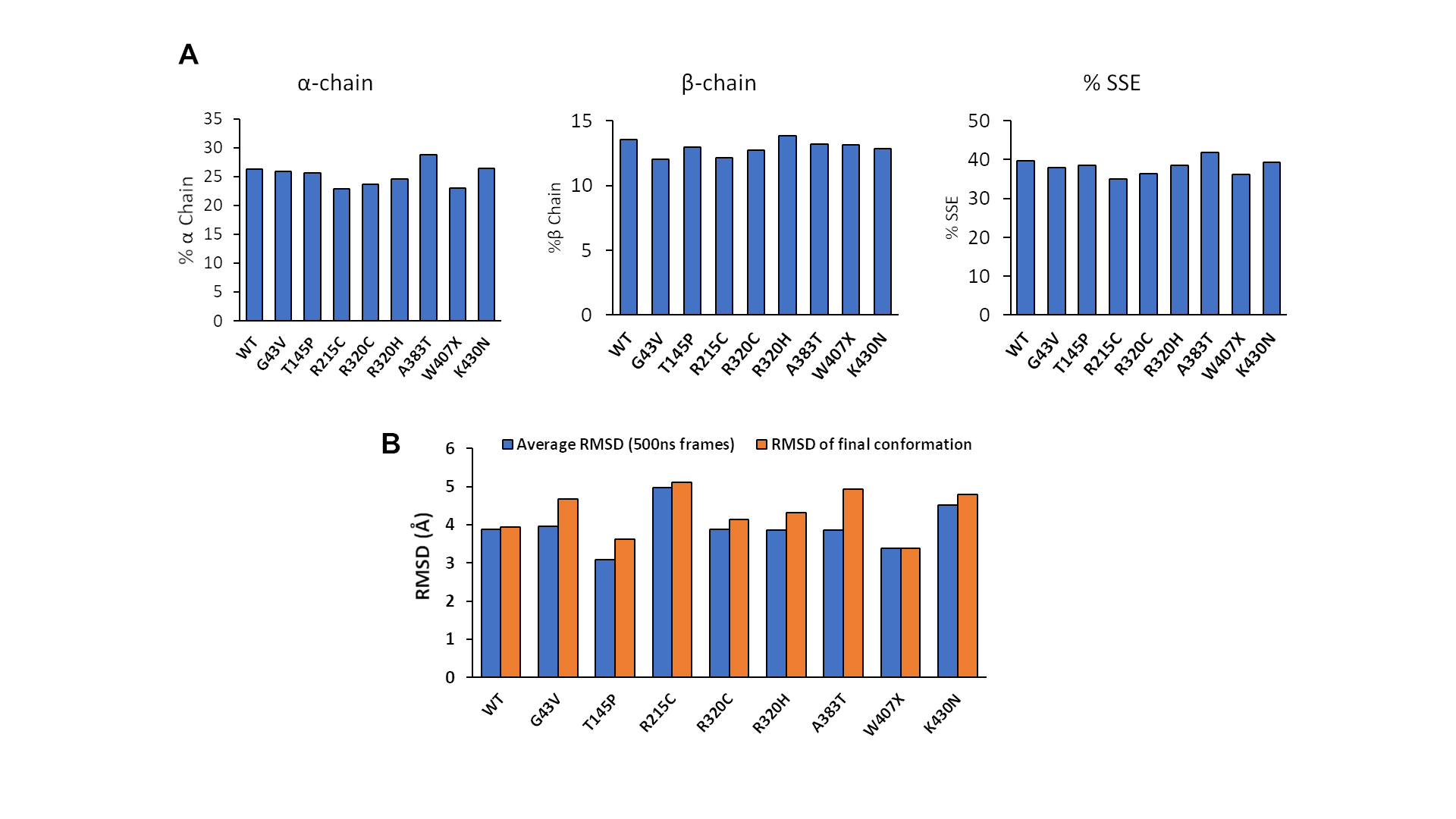

Supplement: Supplementary file 2 — Supplementary Figure S1. [file 41598_2023_28381_MOESM2_ESM.tiff]

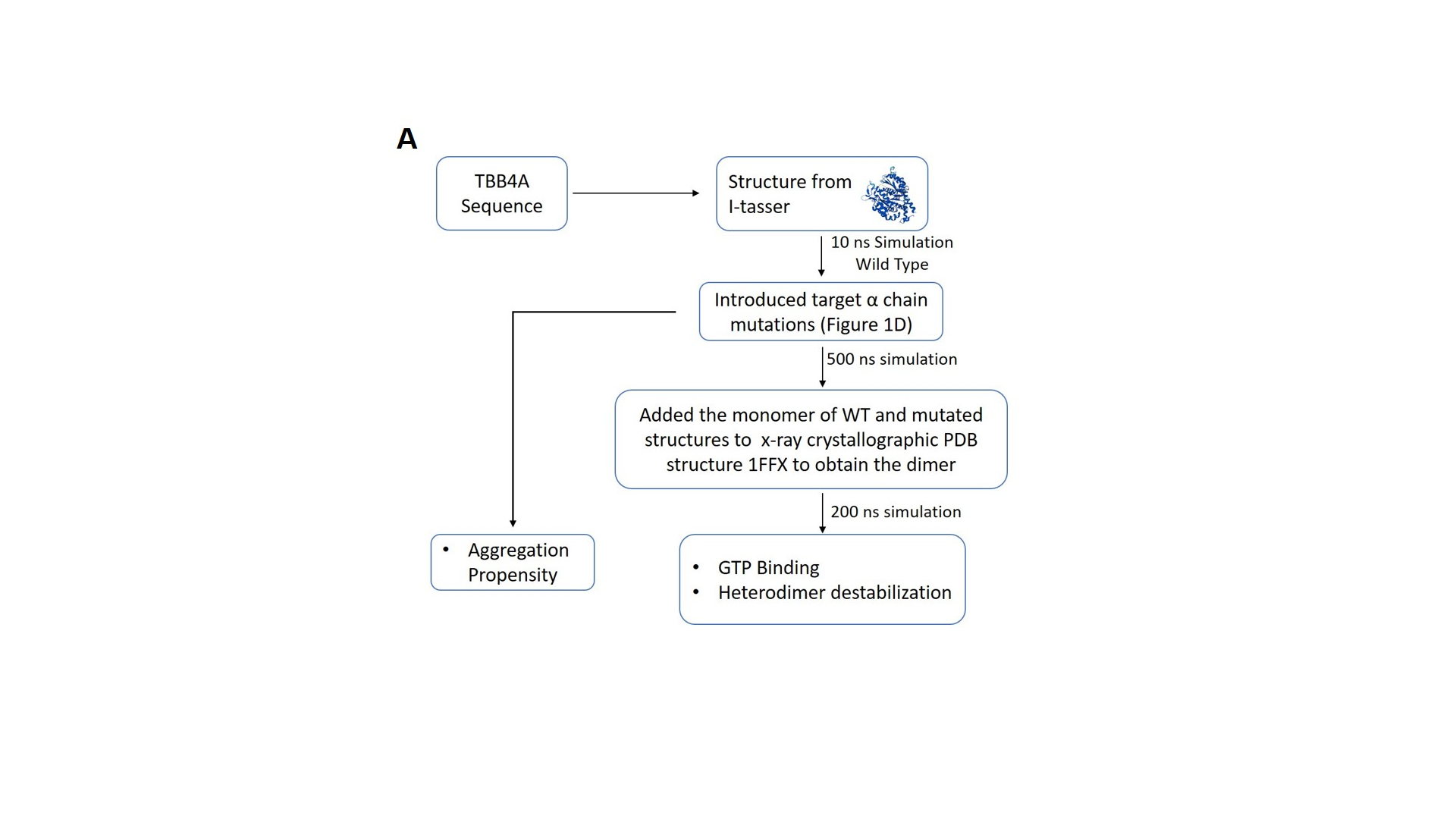

Supplement: Supplementary file 3 — Supplementary Figure S2. [file 41598_2023_28381_MOESM3_ESM.tiff]

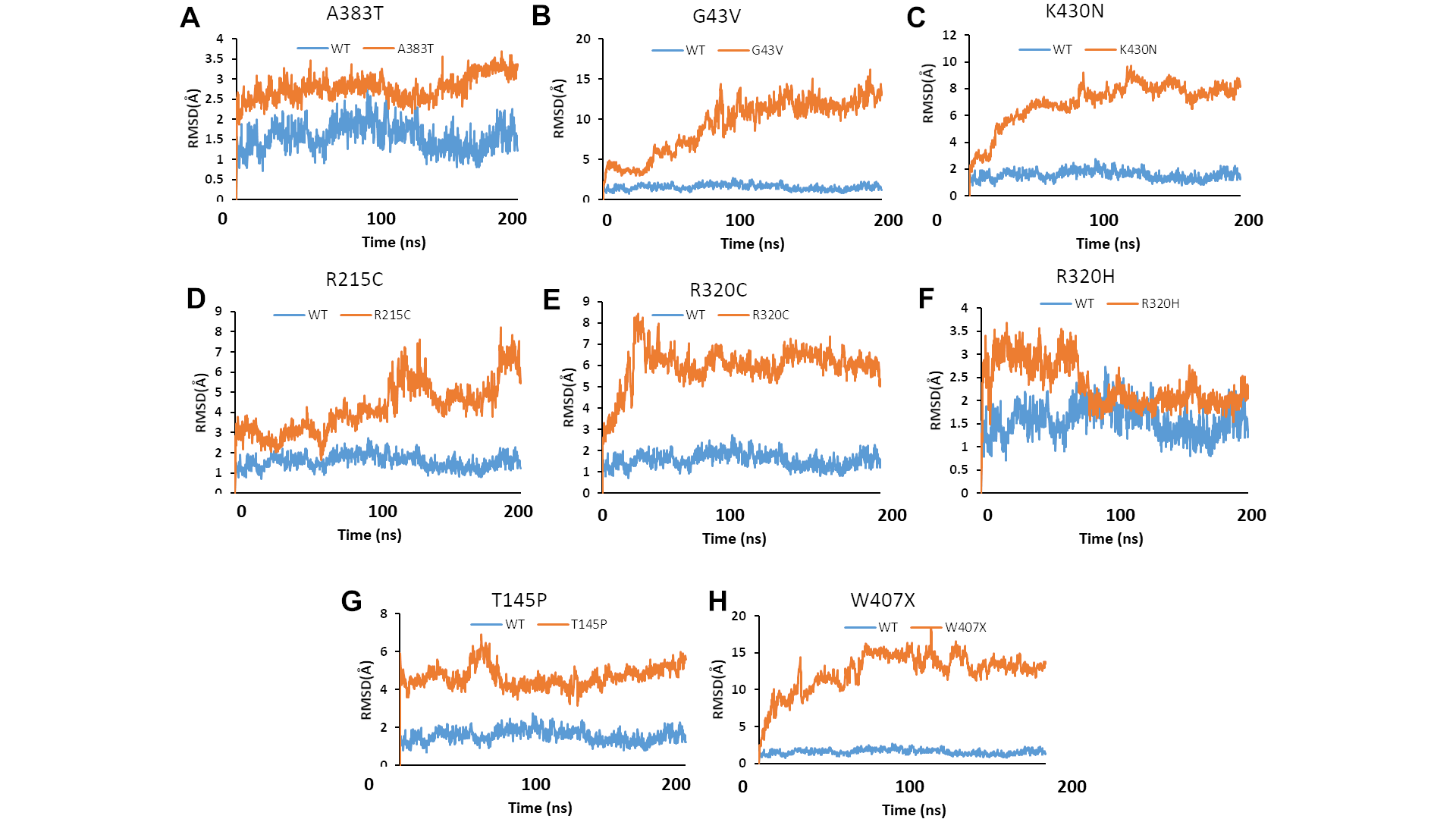

Supplement: Supplementary file 4 — Supplementary Figure S3. [file 41598_2023_28381_MOESM4_ESM.tiff]

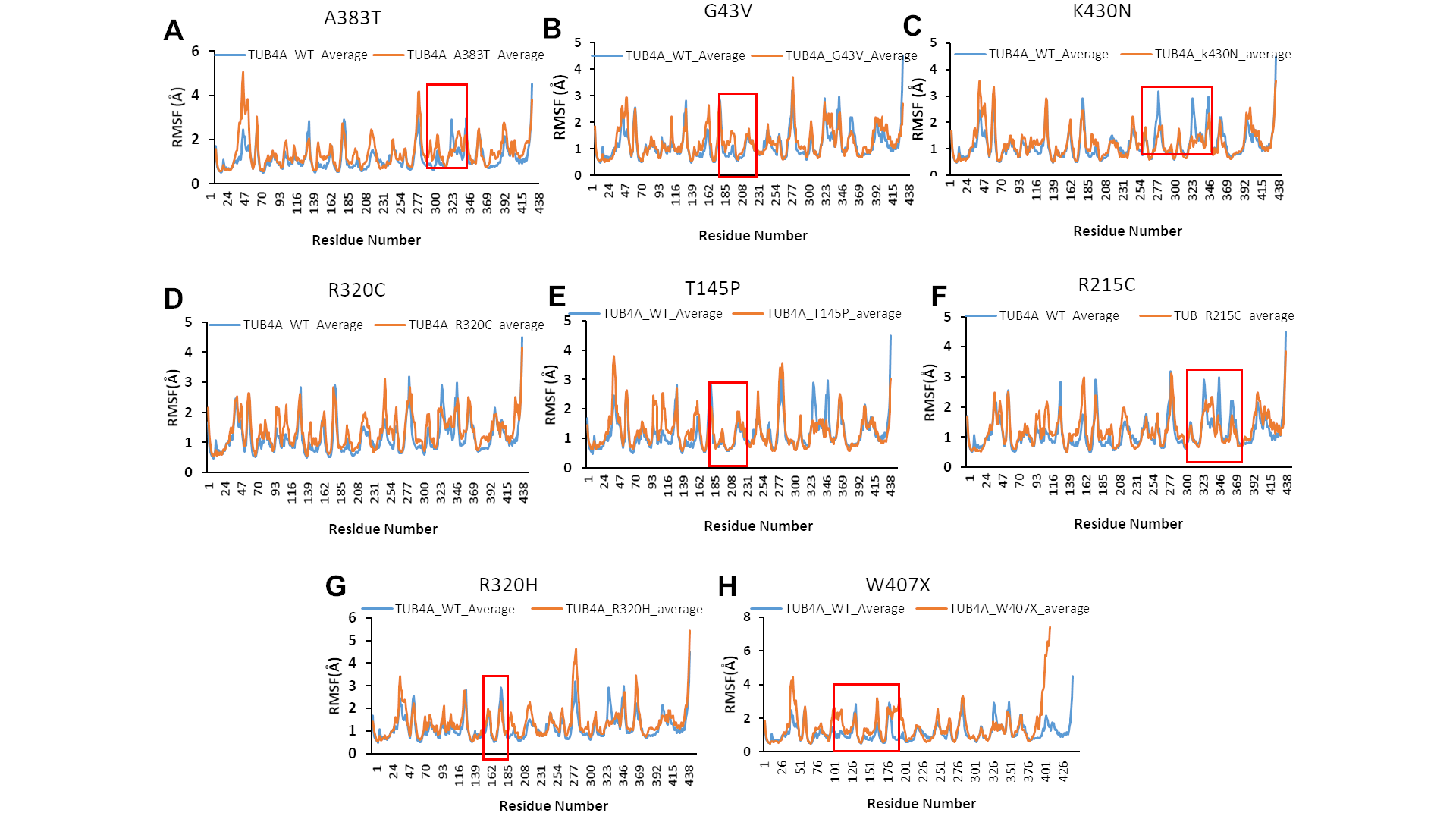

Supplement: Supplementary file 5 — Supplementary Figure S4. [file 41598_2023_28381_MOESM5_ESM.tiff]
